# mSphere of Influence: *Trypanosoma cruzi* genome in 3D action

**DOI:** 10.1128/msphere.00611-24

**Published:** 2025-02-26

**Authors:** Luisa Berná

**Affiliations:** 1 Laboratorio de Interacciones Hospedero-Patógeno-UBM, Institut Pasteur de Montevideo 123939 , Montevideo, Uruguay; 2 Laboratorio de Apicomplejos, Institut Pasteur de Montevideo 123939 , Montevideo, Uruguay; 3 Unidad de Bioinformática, Institut Pasteur de Montevideo, Montevideo, Uruguay; 4 Laboratorio de Genómica Evolutiva, Facultad de Ciencias, Universidad de la República 124158 , Montevideo, Uruguay; Institut Pasteur de Montevideo, Montevideo, Uruguay

**Keywords:** three-dimensional genome organization, nuclear territories, *Trypanosoma cruzi*, chromatin architecture

## Abstract

Luisa Berná works in the field of comparative and evolutionary genomics in unicellular eukaryotes. In this mSphere of Influence article, she reflects on how advances in three-dimensional genome organization have reshaped our understanding of parasite biology. She discusses how recent findings uncover the distinctiveness of the three-dimensional architecture of *Trypanosoma cruzi’*s genome and its functional implications. Berná argues that integrating structural genomics into parasite research is essential for advancing our understanding of genome organization and its role in shaping parasite biology, particularly in the context of neglected tropical diseases.

## COMMENTARY

The advances in the understanding of genetic material—its role in encoding and modulating the cellular forms and functions of all living organisms as well as its structure, compaction, and different organizational levels—reveal an intuitive and almost inevitable need for a sophisticated three-dimensional organization. This organization comprises nuclear territories, loops, and chromatin domains and adds a layer of regulation to the genome function and variability. Surprisingly, the notion of such a structured nuclear organization emerged long before the modern tools that have now confirmed its existence.

The concept of chromosome territories was first suggested by Carl Rabl in 1885 and later expanded by Theodor Boveri, one of the founders of cytology, who formalized the idea of chromosomal territories (along with co-developing the chromosomal theory of inheritance with Sutton) in the early 1900s. However, it was not until the 1970s that a series of elegant experiments directly demonstrated that chromosomes do not randomly coil inside the nucleus like spaghetti in a bowl, but instead, they occupy specific nuclear territories, with defined interactions among them (1, 2). This story rounds up in the 1980s, when *in situ* hybridization techniques allowed the direct visualization of these territories (3, 4).

Although initial studies focused exclusively on large genomes due to technical challenges, subsequent work extended these findings to model organisms holding smaller genomes. Recently, interest has expanded to include the three-dimensional genome organization of non-model organisms, particularly pathogenic species relevant to human and animal health.

One compelling example is *Trypanosoma cruzi*, the causative agent of Chagas disease, a neglected tropical disease that predominantly impacts impoverished populations in Latin America. While there is a long history of research on *T. cruzi*, Chagas disease, and its insect vector in the region, progress in certain areas of study has been slower for this parasite due to its intrinsic biological complexity. For instance, despite *T. cruzi*’s relatively small genome (~55 Mb), its highly repetitive nature, with extensive multigene families containing hundreds of genes, has made it particularly challenging to obtain telomere-to-telomere reference genomes.

Recently, advanced sequencing technologies have provided more complete and accurate *T. cruzi* genomes (5–7). Our work published in 2018, revealed that *T. cruzi’*s nuclear genome organizes into two distinct compartments in terms of compositions, evolutionary rates, and dynamics. One of these compartments, the “core compartment,” is conserved, syntenic, and shared with other trypanosomatids (like *Trypanosoma brucei* or *Leishmania*). In contrast, the “disruptive compartment,” unique to *T. cruzi*, comprises hundreds of copies of genes belonging to surface multigene families critical for host-parasite interaction and immune evasion (5).

The original observation that *T. cruzi’*s linear genome was compartmentalized inherently suggested the existence of a corresponding three-dimensional compartmentalization. This organization would represent an additional regulatory layer, enabling specific interactions and influencing chromatin conformation, accessibility, transcriptional activation, and repression. Indeed, studies have revealed that nuclear morphology changes, epigenetic marks, and chromatin remodeling regulate gene expression encompassing the parasite’s differentiation (8, 9).

The advent of 3C and Hi-C techniques has enabled the capture of physical interactions between distant chromatin regions and the mapping of three-dimensional genome organization. Díaz-Viraqué and colleagues (10) were able to apply this technology to the study of *T. cruzi*’s genome, revealing for the first time that *T. cruzi*’s genome exhibits a distinctive three-dimensional organization.

By analyzing intra-chromosomal interactions, the authors demonstrated that *T. cruzi*’s linear genome compartmentalization is closely linked to its three-dimensional distribution. Core regions of a chromosome preferentially interact with each other, while disruptive regions exhibit higher interaction frequencies, accompanied by denser nucleosome packing and well-positioned nucleosomes. This proximity and high compaction of disruptive regions appear to play a crucial role in facilitating DNA recombination. Such recombination, observed at higher rates in these regions, likely serves as a mechanism underlying antigenic variation—a key strategy employed by trypanosomes to evade host’s immunity.

The study further revealed that the *T. cruzi* genome is organized into chromatin-folding domains, which resemble the well-known topologically associating domains of vertebrates but are significantly smaller (~130 Kb) and exclusively composed of either core or disruptive regions. These domains are particularly compact in the disruptive compartment, as also evidenced by previous FAIRE-seq experiments (8), and likely compensate for the absence of enhancer-promoter regulation, enabling precise control of gene expression. Indeed, by analyzing gene expression across two life stages, Diaz-Viraqué and colleagues showed that this compaction facilitates transcriptional modulation. Most genes in the disruptive compartment remain nearly silent, with only a few activated in trypomastigotes, the bloodstream infective stage. This finding suggests that gene expression in the disruptive compartment is influenced by its three-dimensional genome organization, challenging the paradigm that *T. cruzi* gene expression relies solely on basal transcription followed by post-transcriptional regulation.

Building on these advances, much remains to be uncovered about the organization of the *T. cruzi* genome. Advanced technologies, such as PacBio HiFi, are needed to achieve telomere-to-telomere assemblies, which are fundamental for comprehensive studies of three-dimensional genome organization. Such assemblies would also help determine the number of chromosomes in this species and assess whether this number varies among lineages, as has been proposed. Furthermore, Hi-C data from the infective stage could shed light on chromatin interactions regulating gene expression, while emerging tools like Pore-C, particularly suited for repetitive genomes and complex architectures, may reveal haplotype-specific interactions. This is particularly relevant in *T. cruzi*, where the haplotypes of the disruptive compartment are highly divergent, with roles that remain only partially understood. Investigating these aspects presents a unique opportunity to deepen our understanding of the parasite’s genome architecture and its biological and evolutionary implications within the context of species diversity.

Given the importance of *T. cruzi* for public health and the long tradition of research on this parasite in Latin America, the region’s active and collaborative scientific community is well positioned to address these aspects. Through new partnerships and continued contributions, it is likely that still-unknown questions about the genome and functional organization of this complex parasite will soon be uncovered.
